# Immunity of Nurslings to Certain Infectious Diseases

**Published:** 1911-12

**Authors:** G. Variot

**Affiliations:** La Clinique Infantile


### Immunity of Nurslings to Certain Infectious Diseases

By DR. G. VARIOT, in *La Clinique Infantile*

IT is a well known fact that the resistance of nurslings to the morbid germs of most of the infectious diseases, which act vigorously, is very intense among children two years of age. It is rare to find children less than a year old in tents where the contagious diseases, measles, scarlet fever, diphtheria, etc., are isolated.

Dr. G. Variot from cases occurring in the Hospine dépositaire des Enfants assistés and other institutions of Paris, has collected and is collecting interesting data on the morbidity of the new-born. In the former institution are confined more than 2,000 children less than one year old, who have been either abandoned by their parents, or in temporary trust.

### MEASLES.

Dr. G. Variot cites several cases observed by different men, among whom are Vogel and Sydenham. They found that the nursling, kept to its room and imprisoned in its swaddling clothes almost all day, is thus more protected against infection than the four or five year old child.

Another, M. Hutinel, in his treatise on the diseases in childhood, states that the new born is exceptionally affected by contagion. Children now in contact with disease are less often susceptible to it than others. Yet cases are recorded by Sevestre, where nurslings did not contract measles during the infection of their nurses. Immunity of children, is not always absolute; for in an epidemic of measles among nurses who were caring for very young children, all had a light eruption, a very attenuated measles. But the transmission of measles is rarer than smallpox.

STATISTICS ON THE DISEASE.

At the Children's Hospital of Chatillon, Sous Bagneux; not one case of measles was observed in the last 4 years.

At the Pasteur tent; out of 241 children under one year only two cases of measles were observed: one 5 months old, which died, and the other 10 months old.

The total number of cases of measles among nurslings up to one year of age, admitted to isolated tents during the last 4 years:

1908—1 measles, 6 months, cured; 1 measles, bronchopneumonia, 11 months, died.

1909—1 measles, 11 months, cured; 1 measles, bronchopneumonia, 9 months, died.

1910—1 measles, bronchopneumonia, 7 months, died; 1 measles, 10 months, cured.

1911—1 measles, 8 months, cured.

The general total of cases of measles is about 1,375 for these 4 years, and are distributed as follows:

Age, 1-2 years—282 cases.

Age, above 2 years—1086 cases.

Age, below 1 year—7 cases.

SCARLET FEVER.

Again, Billard believed that scarlet fever is more prevalent in late childhood and in adolescence than among nurslings. He saw during the year 1826, three children from one year to 15 months old, seized with scarlet fever, while not one of the younger children were affected.

In the great epidemics nurslings are almost always spared. Thus in that of Saalbourg (1785), Washington (1827), Halle 1818), Hanau (1819), the children did not contract the disease (Virot). However rare scarlet fever may be in early childhood, it is nevertheless sometimes observed among the new born (Ferrario, Tortual, Portier).

Welch and Schaumberg of Philadelphia, in their treatise "Acute Contagious Diseases," say that age is a very important factor in susceptibility to scarlet fever. Children under one year show to a lesser degree, a predisposition to contract scarlet fever. This is more true of children under six months, and among children under three months, scarlet fever is extremely rare. They have found that out of 5,000 cases of scarlet fever, admitted to the municipal hospital of Philadelphia, about 1 per cent. concerns children less than 1 year old.

Out of the 1,272 nurslings sent out to the Children's Hospital of Chatillon, only one case of scarlet fever was observed in four years.

There has been no more than one case under one year in the tents for contagious diseases, out of 164 cases of scarlet fever.

